# Impedance-Based Living Cell Analysis for Clinical Diagnosis of Type I Allergy

**DOI:** 10.3390/s17112503

**Published:** 2017-10-31

**Authors:** Reiko Irifuku, Yuhki Yanase, Tomoko Kawaguchi, Kaori Ishii, Shunsuke Takahagi, Michihiro Hide

**Affiliations:** Department of Dermatology, Graduate School of Biomedical and Health Science, Hiroshima University, 1-2-3 Kasumi, Minami-ku, Hiroshima 734-8551, Japan; toku_risu@yahoo.co.jp (R.I.); yyanase@hiroshima-u.ac.jp (Y.Y.); tomokok@hiroshima-u.ac.jp (T.K.); ishiik@hiroshima-u.ac.jp (K.I.); takshuns@gmail.com (S.T.)

**Keywords:** impedance sensor, diagnosis of type I allergy, mast cells, human IgE receptor-expressing cells, IgE antibody, serum, histamine release test

## Abstract

Non-invasive real time evaluation of living cell conditions and functions are increasingly desired in the field of clinical diagnosis. For diagnosis of type I allergy, the identification of antigens that induces activation of mast cells and basophils is crucial to avoid symptoms of allergic diseases. However, conventional tests, such as detection of antigen-specific IgE antibody and skin tests, are either of low reliability or are invasive. To overcome such problems, we hereby applied an impedance sensor for label-free and real-time monitoring of mast cell reactions in response to stimuli. When IgE-sensitized RBL-2H3 cells cultured on the electrodes were stimulated with various concentrations of antigens, dose-dependent cell index (CI) increases were detected. Moreover, we confirmed that the impedance sensor detected morphological changes rather than degranulation as the indicator of cell activation. Furthermore, the CI of human IgE receptor-expressing cells (RBL-48 cells) treated with serum of a sweat allergy-positive patient, but not with serum from a sweat allergy-negative patient, significantly increased in response to purified human sweat antigen. We thus developed a technique to detect the activation of living cells in response to stimuli without any labeling using the impedance sensor. This system may represent a high reliable tool for the diagnosis of type I allergy.

## 1. Introduction

**Figure 1:**
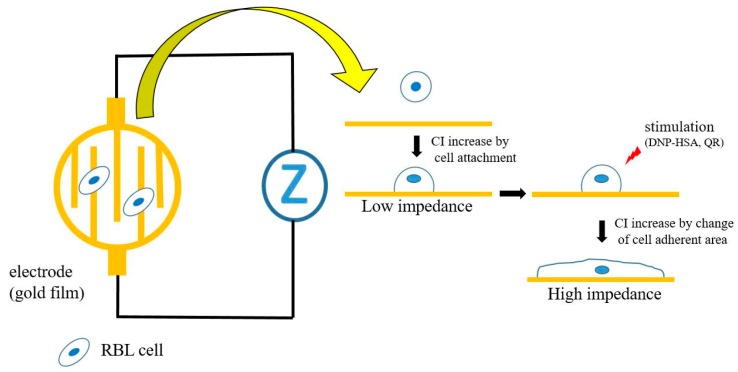
Schematic of the impedance system for living cells. The impedance sensor detects the attachment and morphological change of cells on electrodes.

Since the cell is the minimum unit of living organisms, non-invasive real time observation and the evaluation of living cell conditions and functions are increasingly desired in the field of life science and for clinical diagnosis. Recently, various kinds of biosensors for living cell analyses, such as the quartz crystal microbalance (QCM) sensor [1,2], the field-effect transistor (FET) sensor [3], the surface plasmon resonance (SPR) sensor [4], and the resonant waveguide grating (RWG) sensor [5], have been reported. QCM sensors detect mass, thickness, and viscoelastic properties of living cells on the sensor. FET sensors detect the charge density derived from the living cell activity near a sensor. SPR sensors and RWG sensors detect the dielectric constant of the evanescent field, which penetrates the cells on a sensor. Since SPR sensors detect the refractive index (RI) near the plasma membrane in the SPR detection area (<500 nm), the RI changes detected by SPR sensors reflect various reactions of the cells, such as morphology, membrane potential, and the density of proteins. On the other hand, impedance sensors measure electric impedance between the electrode that is dependent for instance on the area of attachment of cells on the surface of electrodes (Figure 1).

Urcan et al. applied an impedance sensor (xCELLigence™ system) for continuous monitoring of the proliferative capacity of human gingival fibroblasts [6]. Guan et al. developed and evaluated a rapid, label-free phenotypic assay for the assessment of T cell activation in response to TCR stimulation using the xCELLigence™ system [7]. It is well known that mast cells residing in tissue and basophils circulating in peripheral blood play important roles in diseases and/or conditions driven by type I allergy, such as asthma, allergic rhinitis, urticaria, and anaphylactic shock. With respect to immunoglobulins, which are involved in immune reactions, there are five main classes of heavy chain constant domains. Each class defines the IgM, IgG, IgA, IgD, and IgE isotypes [8]. Mast cells and basophils express the high-affinity IgE receptors (FcεRI) on their cell surface, and IgE antibodies in serum bind to the IgE receptors. When specific antigens, such as those in food, mites, and pollen, enter the body and bind to specific IgE antibodies on the cell surface, they crosslink the IgE receptors and activate several tyrosine kinases (TK), such as Lyn and Syk [9]. These kinases then activate other signaling molecules, including phosphatidylinositol 3-kinase (PI3K) and phospholipase Cγ (PLCγ). PLCγ cleaves phosphatidylinositol 4, 5-bisphosphate (PIP_2_) into two crucial second messengers: inositol 1,4,5-triphosphate (IP_3_), a Ca^2+^ releaser from cellular stores, and diacylglycerol (DAG), an activator of protein kinase C (PKC). IP_3_ induces the depletion of Ca^2+^ stores, which in turn activates Ca^2+^ release-activated Ca^2+^ (CRAC) channels and causes capacitative Ca^2+^ entry [9]. These Ca^2+^ responses, which are followed by the activation of PKC, induce the release of various chemical mediators, such as histamine, from mast cells and basophils, and allergic reactions (Figure S1). IgE receptor-dependent activation of mast cells also causes dynamic polymerization and reorganization of the actin cytoskeleton, ruffling of the plasma membrane, and spreading of the cell, which appear to play important roles for the amplification of allergic reactions. Since these functions are also regulated by the TK-PLC-PKC signal transduction pathway, the degree of morphological change of mast cells and basophils in response to an allergen are proportional to that of degranulation. Therefore, it is very important to detect specific antigens (also called allergens) and/or the sensitivity of IgE to the antigens, which induce allergic reactions in each patient. Various diagnostic tests for type I allergy, such as the detection of serum IgE, histamine release from basophils, or skin reactions to allergens, are performed [10,11,12,13,14,15,16,17]. In order to detect antigen-specific IgE in serum, ImmunoCAP^TM^, Ala-STAT^TM^, and AD-VIA Centaur^TM^ have been developed and utilized in clinical practices [10]. However, there are often substantial discrepancies between the data of serological tests and clinical symptoms [10,11]. Both mast cells and basophils, which are sensitized with the same repertoire of IgE in individual subjects, release various substances, including histamine, in response to the antigen (Figure S1). Basophils also express several molecules on their surface, such as CD203c and CD69, which are used as activation markers. Therefore, the basophil histamine release test (HRT) and basophil activation test (BAT) for the detection of upregulation of CD203c or CD69 by flow cytometry are sensitive, safe, and offer reliable information regarding antigen that causes type I hypersensitivity in vitro [12,13]. Griese et al. reported that the histamine release test showed higher sensitivity and specificity than the skin test or serum analysis, such as ImmunoCAP^TM^, based on the comparison with bronchoprovocation of extrinsic asthmatic children [14]. Moreover, BAT based on CD203c upregulation has been validated as a reliable tool for the diagnosis of IgE-mediated allergies [15]. However, HRT requires a substantial quantity of blood for multiple tests, making such testing impractical to perform for babies and infants in clinical practices. With respect to its limitations, BAT requires expensive analytical equipment, such as flow cytometry, and needs sustainable maintenance of fluidic channels. Furthermore, HRT and BAT require fresh peripheral blood collected within a few days. In vivo tests, such as skin tests and antigen challenge tests, are more reliable in reflecting clinical conditions. However, these tests may be painful and could potentially evoke anaphylactic shock when a patient is extremely sensitive to a particular antigen [16]. Moreover, the intradermal injection of an antigen may sensitize subjects who are not sensitized to the particular antigen [16].

To overcome these problems in the diagnosis of type I allergy, we established a system to monitor the adhesion area of surrogate cells, RBL-48 cells, a human IgE receptor-expressing mast cell line, sensitized with human serum IgE in response to antigen by means of the impedance sensor. We also confirmed its potential for the application to the clinical diagnosis of type I allergy.

## 2. Materials and Methods

### 2.1. Reagents

Chemicals were obtained from the following sources: bovine serum albumin (BSA), dinitro-phenol-conjugated human serum albumin (DNP-HSA), DNP-specific rat monoclonal IgE and Phalloidin-TRITC from Sigma-Aldrich Japan (Tokyo, Japan); anti-IgE from BETYL (Montgomery, TX, USA); fetal calf serum (FCS) from Biowest (Nuaillé, France); penicillin/streptomycin, trypsin, and G418 from Life Technologies (Carlsbad, CA, USA); phorbol 12-myristate 13-acetate (PMA), ionomycin, genistein, cytochalasin D and nocodazole from Calbiochem (San Diego, CA, USA); epidermal growth factor (EGF) from R&D Systems (Minneapolis, MN, USA); DAPI from Dojindo (Kumamoto, Japan).

### 2.2. Cell Lines

RBL-2H3 cells were cultured in RPMI medium supplemented with 10% fetal calf serum (FCS), 100 U/mL penicillin, and 100 μg/mL streptomycin. On the day before experiments, cells were harvested using trypsin and then cultured (1 × 10^5^ cells/mL) in glass bottom culture dishes for microscopic observation, 96-well plate for a degranulation assay or E-plate for impedance measurement. The RBL-48 cell line was derived from an RBL-2H3 cell line transfected with the human α-subunit of FcεRI, kindly supplied as a gift by Dr. Joko Kochan (Hoffmann- La Roche, Nutley, NJ, USA) and cultured in IMDM medium supplemented with 10% fetal calf serum (FCS), 100 U/mL penicillin, 100 μg/mL streptomycin, and 0.5 mg/mL G418 [17]. On the day before experiments, RBL-48 cells were harvested using trypsin and then cultured in a glass bottom culture dishes (2 × 10^5^ cells/mL) and E-plates in the presence of human serum in a ratio of cell suspension to serum of 30:1. The volume of cell suspension in a well of E-plate was 200 μL (2 × 10^5^ cells/mL). PAM212 cells were grown in DMEM supplemented with 10% FCS, 100 U/mL penicillin, and 100 μg/mL streptomycin.

### 2.3. Impedance Measurement

We used iCELLigence™ (ACEA Biosciences, Inc., San Diego, CA, USA) as the impedance sensor for the monitoring of living cell reactions with E-Plate (8 wells), which has comb-shaped electrodes on the bottom of each well (Figure 1). The assay system expresses impedance in an arbitrary cell index (CI) units (Rn–Rb)/4.6; where Rn is the cell–electrode impedance of the well when it contains cells and Rb is the background impedance of the well with the media alone. The frequency for impedance detection is 10 kHz, which is suitable for detection of the adhesion area of living cells on the electrodes (product information of iCELLigence™). Obtained changes of CI were analyzed with RTCA data analysis software (ACEA Biosciences). RBL-2H3 cells or RBL-48 cells were cultured on an E-plate with or without human serum collected from peripheral blood. The sensor well, on which living cells were cultured, was placed on iCELLigence™ in a CO_2_ incubator at 37 °C. The change of impedance on the electrodes in each well was then monitored. 

### 2.4. Assays of Mast Cell and Basophil Degranulations (Release of β–Hexosaminidase and Histamine)

Degranulation of RBL-2H3 cells and RBL-48 cells was evaluated 15 min after stimulation with the release of β-hexosaminidase, a granule marker, by the hydrolysis of p-nitropheny-N-acetyl-β-glucosamine to the chromatophore, p-nitrophenol as described previously [18]. Histamine release from human basophils was measured as reported previously [19].

### 2.5. Subjects

Age, sex, and sensitivity of each donor are shown in Table 1.

### 2.6. Actin Cytoskeleton and Nuclei Staining, and Calculation of the Area of Cell Attachment

RBL-2H3 cells and RBL-48 cells were fixed with 4% paraformaldehyde 10 min after stimulation in glass bottom dishes. Following two PBS washes, the cells were treated with PBS containing phalloidin-TRITC for actin-staining and DAPI for nuclei-staining for 30 min. Cells on the glass-bottomed dishes were photographed under a fluorescent microscope, and their images analyzed by computerized conversion of the pixels using Image-Pro^®^ Plus (Media Cybernetics, Inc., Rockville, MD, USA). The values described here indicate the mean of the area of 8–15 randomly sampled cells.

### 2.7. Measurement of QR-Specific Antibodies

We performed an enzyme-linked immunosorbent assay (ELISA) for QR-specific IgE according to a method which has been described previously [20].

## 3. Results

### 3.1. Real-Time and Label-Free Monitoring of Living Cell Activations by Means of Impedance Sensor

**Figure 2:**
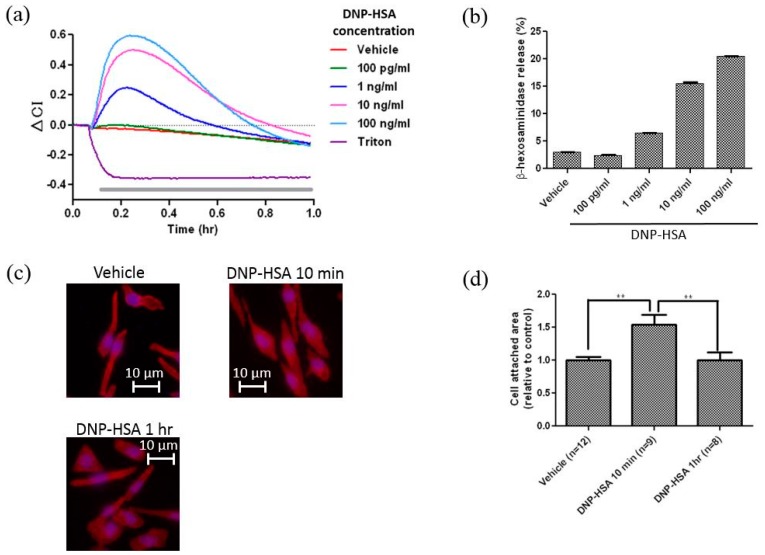
(**a**) CI changes (ΔCI) of RBL-2H3 cells in response to various concentrations of antigen. The gray bar indicates the presence of DNP-HSA or Triton. CI first increased in a DNP-HSA concentration-dependent manner. Maximum CI changes were observed around 10 min after the stimulation. CI, then, decreased to baseline in around 1 h. (**b**) Degranulation of RBL-2H3 cells in response to various concentrations of antigen. Degranulation of RBL-2H3 cells was evaluated with release of β-hexosaminidase, a granule marker, by measuring the hydrolysis of p-nitropheny-N-acetyl-β-glucosamine to the chromatophore, p-nitrophenol. The graph is representative of three experiments. Data were obtained from quadruplicate measurements. (**c**) Changes of the adhesion area of RBL-2H3 cells in response to DNP-HSA. Red shows actin cytoskeleton stained by phalloidin-TRITC, and blue shows nuclei stained by DAPI. White bar shows ca. 10 μm. (**d**) The relative cell area of RBL-2H3 cells before and after stimulation by DNP-HSA. The adhesion area of each cell was measured by using Image-Pro Plus 6.3J (Media Cybernetics, Bethesda, MD, USA). The difference between the area of cells among each group was statistically analyzed using a one-way analysis of variance followed by Tukey’s test (** *p* < 0.01). ‘n’ means the number of cells measured.

We first determined whether the sensor could detect the activation of RBL-2H3 cells, which are widely used as cognate mast cells, in response to stimuli. RBL-2H3 cells were cultured overnight on the electrodes at the density of 1.0 × 10^5^/mL (200 μL/well) with anti-DNP IgE (50 ng/mL). When IgE-sensitized RBL-2H3 cells on the electrodes were stimulated with various concentrations of DNP-HSA, CI increased in a dose-dependent manner (Figure 2a). Maximum changes of CI were observed 10 min after stimulation (Figure 2a). Similar results were obtained with a β-hexosaminidase assay [4], which shows the degree of degranulation of RBL-2H3 cells (Figure 2b). Figure 2 shows RBL-2H3 cells stained with phalloidin-TRITC (for actin cytoskeletons) and DAPI (for nuclei) (c), and the area of cell adhesion calculated by these images (d). The area of actin cytoskeleton increased after a stimulation and reached maximum around 10 min after the stimulation (Figure 2c,d). These results suggest that the kinetics of impedance changes caused by RBL-2H3 cells activation is similar to the kinetics of histamine release and morphological changes caused by actin cytoskeletons. We then treated cells with 0.1% TritonX-100 buffer, which dissolves the lipid bilayer of the plasma membrane, leaving adhesion molecules on the sensor chip. In response to this treatment, CI quickly decreased, suggesting that impedance caused by living cells on electrodes largely reflects binding of plasma membrane to the electrodes (Figure 2a). In order to confirm if the system can detect the activation of other types of cells, we monitored the reaction of a mouse keratinocyte cell line, PAM212 cells, in response to epidermal growth factor (EGF) [21]. When PAM212 cells were cultured on electrodes overnight at the density of 4.0 × 10^5^/mL (200 μL/well) and stimulated with various concentration of EGF, CI quickly increased for 5 min and then gradually decreased to, but not below, the baseline (Figure S2). 

**Figure 3:**
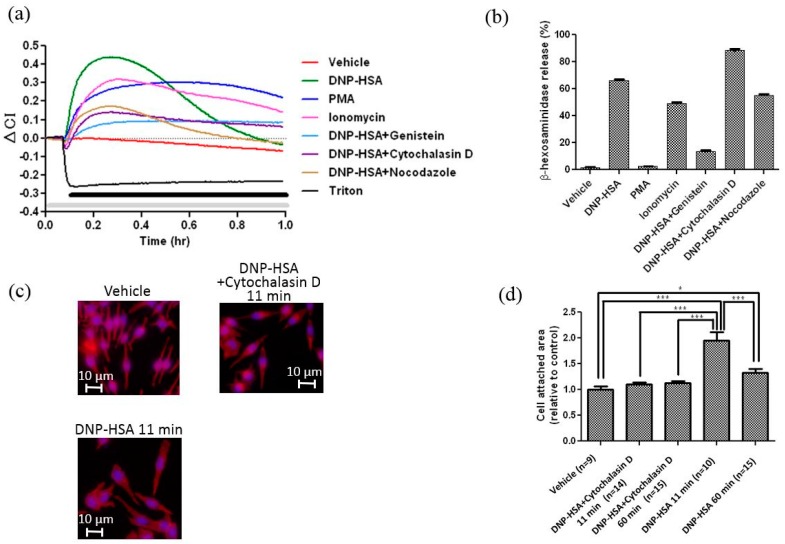
(**a**) Effects of activators (DNP-HSA 50 ng/mL, PMA 50 nM, Ionomycin 1 μM) and inhibitors (Genistein 100 μM, Cytochalasin D 1 μM, Nocodazole 10 μM) on CI changes (ΔCI) of RBL-2H3 cells. The gray bar indicates the presence of each inhibitor, and the black bar indicates the presence of antigen, activator, or Triton. (**b**) Effects of activators and inhibitors on degranulation of RBL-2H3 cells. The graph is representative of three experiments. Data were obtained from quadruplicate measurements. (**c**) Effects of Cytochalasin D on cell spreading in response to antigen. The white bar shows ca. 10 μm. (**d**) Relative cell area of RBL-2H3 cells before and after stimulation by DNP-HSA with or without Cytochalasin D. The area of cell adhesion was measured by using Image-Pro Plus 6.3J. Differences between the areas in each group were analyzed using one-way analysis of variance followed by Tukey’s test (* *p* < 0.05, *** *p* < 0.001). ‘n’ means number of cells measured.

We next investigated the effect of various kinds of activators (PMA and ionomycin) and inhibitors (genistein, cytochalasin D, and nocodazole) on the CI change of RBL-2H3 cells (Figure 3a). PMA (PKC activator) and ionomycin (Ca^2+^-ionophore) increased the CI of RBL-2H3 cells, suggesting that protein kinase C (PKC) and calcium mobilization play an important role for the CI change of living cells (Figure 3a). Genistein (tyrosine kinase inhibitor) strongly inhibited an antigen-induced CI increase and degranulation (Figure 3a,b), suggesting that the activation of intracellular signal transduction, such as tyrosine phosphorylation, is required for CI changes of RBL-2H3 cells detected by the impedance sensor. Cytochalasin D (actin polymerization inhibitor) and nocodazole (microtubule polymerization inhibitor) partially blocked antigen-induced CI increase (Figure 3a). On the other hand, PMA induced a substantial level of slow and prolonged increase of CI, whereas it did not induce degranulation by itself (Figure 3a,b). We also studied the effects of inhibitors on CI of RBL-2H3 cells and its changes (ΔCI) in response to DNP-HSA (Figure S3). Since genistein blocks constitutional signal transduction for cytoskeleton rearrangement, and cytokeratin and nocodazole directly affect cytoskeleton rearrangement, CI decreases of RBL-2H3 cells in response to these inhibitors without stimulation were observed. It is noteworthy that cytochalasin D largely enhanced antigen-induced degranulation (Figure 3b), while it partially suppressed both the CI increase (Figure 3a) and cell spreading induced by the antigen (Figure 3c,d). These results suggest that the impedance sensor detects horizontal cell spreading rather than degranulation induced by the activation in RBL-2H3 cells. 

### 3.2. Clinical Diagnosis of Type I Allergy with Serum by Means of Impedance Sensor and RBL-48 Cells

**Figure 4:**
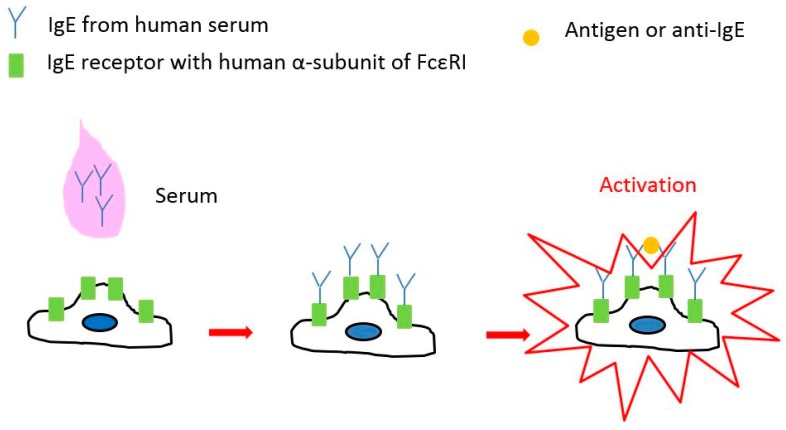
Schematic of RBL-48 cells activation. Serum IgE antibodies in a patient bind to IgE receptors on the surface of RBL-48 cells. Cross-linkage of IgEs by anti-human IgE antibodies or multivalent antigens induces the activation of RBL-48 cells.

We previously reported that RBL-48 cells, an RBL-2H3 cell line expressing the α-subunit of human FcεRI, may be sensitized with human IgE and activated in response to anti-human IgE antibody (anti-IgE) or specific antigens, which induce allergic reactions in donors of serum IgE (Figure 4) [19]. 

To confirm the potential of impedance analysis of the cells as a technique for diagnosis of type I allergy, we cultured RBL-48 cells on the electrodes of iCELLigence™ with the sera of various donors. We then stimulated cells with anti-IgE and sweat antigen, QR, which is known as a major antigen associated with atopic dermatitis [22]. The sensitivities of donors to QR are shown in Table 1.

**Figure 5:**
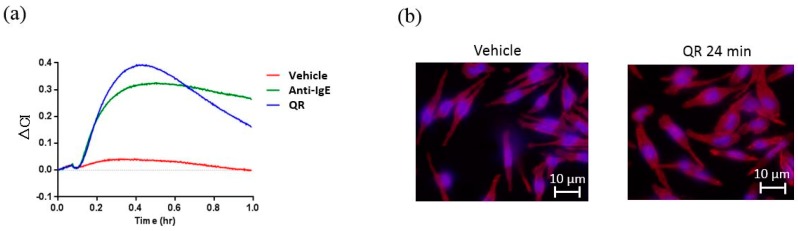

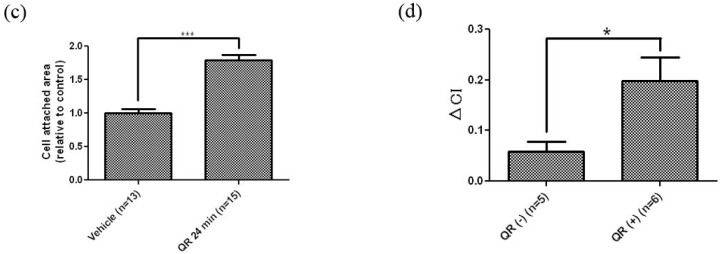
(**a**) Real-time monitoring of CI of RBL-48 cells treated with serum from donor 1 with or without stimulation (RBL-48 cells in 194 µL suspension and 6 µL human serum per well). ΔCI of cells increased in response to anti-IgE or QR. Maximum changes were observed around 24 min after the stimulation. (**b**,**c**) Horizontal spreading of RBL-48 cells in response to QR. The white bar shows ca. 10 μm. Cell area was measured by Image Pro. ‘n’ means number of cells measured. (**d**) Average CI change (ΔCI) of RBL-48 cells treated with serum of QR-positive and that with serum of QR-negative patient in response to QR (*; <0.05). Error bars represent standard error.

As shown in Figure 5a, CI increased in response to anti-IgE or QR. The area of cell adhesion detected by actin staining also increased in response to QR (Figure 5b,c). Figure 5d shows that the CI of RBL-48 cells sensitized with the sera of QR-positive patients, but not with the sera of QR-negative patients, increased in response to QR (Figure 5d). Thus, the impedance sensor can detect the activation of RBL-48 cells, sensitized with the specific IgE of patients, in response to allergen. The amounts of ∆CI in response to QR were also compared with serum concentration of QR-specific IgE measured via ELISA. The levels of QR-specific IgE measured by the two methods showed a weak correlation, suggesting the reliability of the cell-based impedance sensor (Figure S4). Only a weak CI increase was detected in RBL-48 cells sensitized with the serum of a patient who showed a high level of QR-specific IgE by ELISA. Comparisons of precise sensitivity and the specificity of the two assays needs to be assessed by a large-scale clinical study.

## 4. Discussion

In this study, we established living cell reaction-based diagnostic test of type I allergy with a small sample of conserved patient’s serum (6 µL per well) using an impedance sensor. Conventional tests, such as the histamine release test, require a relatively large amount of blood (>1 mL). However, less than one milliliter of blood (300–500 µL of serum) is enough for testing using the system developed in this study. Specific IgE-antibody detection tests, such as IgE binding assays require relatively small amount of serum, but detect only the presence of IgE molecules that bind to an antigen, regardless of their biological activities. Ishii et al. reported a human monoclonal IgE antibody that binds to MGL-1304 in human sweat, but does not activate human basophils [20]. On the other hand, the technique we developed detects the biological activity of IgE that binds to FcεRI expressed on mast cells and basophils, and activates the cells in response to specific antigens. Basophil activation tests, such as HRT, may also detect basophil activation, in response to specific antigen. However, these tests require fresh blood taken from the patient within 2–3 days after collection, whereas sera to be analyzed by the impedance-based test may be preserved for extended periods of time as frozen samples. Moreover, this system could be applied even for subjects whose basophils do not release histamine in response to stimuli (non-responder), since it detects a change of plasma membrane rather than degranulation of mast cells sensitized with IgE from a patient. In fact, we detected an increase in the CI of RBL-48 cells sensitized with IgE from a non-responder in HRT in response to anti-IgE. These results suggest that our system may be more suitable than basophil activation tests as a tool to evaluate allergies of the patients (Figure S5).

SPR and SPR imaging sensors also detect reactions of living cells without any labeling as the change of RI on the sensor chip (thin gold surface) and may be applied for clinical diagnosis of type I allergy [4,18,23,24,25,26,27,28,29]. However, they are not suitable for high-throughput screening analysis, since they detect changes only in an incident light reflected area (the diameter is 1–10 mm). Impedance sensors cannot visualize the distribution of impedance for single cell analysis, which requires high resolution (less than 1 μm). Thus, impedance sensors may be readily applied for low-volume and high-throughput screening assays (the maximum impedance sensing channels of impedance sensor in xCELLigence RTCA HT™ is 1536 wells). Moreover, the physical parameters measured by SPR sensors and those by impedance sensors are somewhat different. SPR sensors detect RI changes in and around the plasma membrane, which reflects the distribution of living cell lipids, proteins, and ions in the SPR detection area (<500 nm). On the other hand, the impedance sensor employed in this study (working at a frequency of 10 kHz) measures the cell contribution to the impedance between the two electrodes, which is related to its adhesion area, morphology, and microstructure. In fact, the pattern of CI changes of PAM212 cells in response to EGF detected by the impedance sensor was largely different from that of RI changes detected by the SPR sensor (Figure S2) [24]. At present, it is not clear which assay reflects clinical conditions more precisely. The combination of the impedance sensor and the SPR sensor may enable us to perform an entirely new real-time, non-labeling and multi-parametric living cell analysis, which has not been achieved with conventional optical microscopes.

## 5. Conclusions

We developed a technique using an impedance sensor to detect the functional activation of human IgE that binds to and activates FcεRI-expressing cells in response to antigens. The technique may be a useful tool for highly reliable, high-throughput diagnosis testing of type I allergy with a small sample of a patient’s serum. 

## Figures and Tables

**Table 1:** Age, sex, and sensitivity of each donor.

Subjects	Age	Sex	Reaction to QR (Histamine Release Test)	Reaction to QR (β-hexosaminidase Assay)
Donor 1	22	M	Positive	Positive
Donor 2	25	F	Positive	Positive
Donor 3	15	M	Positive	Positive
Donor 4	22	M	Positive	Positive
Donor 5	56	F	Positive	Positive
Donor 6	37	F	Positive	Positive
Donor 7	19	F	Negative	Negative
Donor 8	27	F	Negative	Negative
Donor 9	44	F	Negative	Negative
Donor 10	30	M	Negative	Negative
Donor 11	50	M	Negative	Negative

## Supplementary Materials

The following are available online at http://www.mdpi.com/1424-8220/17/11/2503/s1. Figure S1, the mechanism of type I allergy. Figure S2, Cl changes (ΔCl) of PAM212 cells in response to EGF. Figure S3, effects of inhibitors on CI of RBL-2H3 cells with or without stimulation by DNP-HSA. The gray-bar indicates the presence of each inhibitor, and the bold black-bar indicates the presence of DNP-HSA. Figure S4, correlation of the amount of QR-specific IgE in sera of patients detected by the cell-based assay using an impedance sensor and ELISA and Figure S5, (a) Cl changes of RBL-48 cells sensitized with serum a non-responder in response to anti-IgE. (b) Histamine release of basophils from a non-responder in response to anti-IgE.
