# Supplementary material for: Impedance-Based Living Cell Analysis for Clinical Diagnosis of Type I Allergy
Source: Sensors (Basel). 2017 Oct 31;17(11):2503. doi: 10.3390/s17112503 (PMC5713047; doi:10.3390/s17112503)
Supplement: Supplementary file 1 [file sensors-17-02503-s001.pptx]

## Slide 1
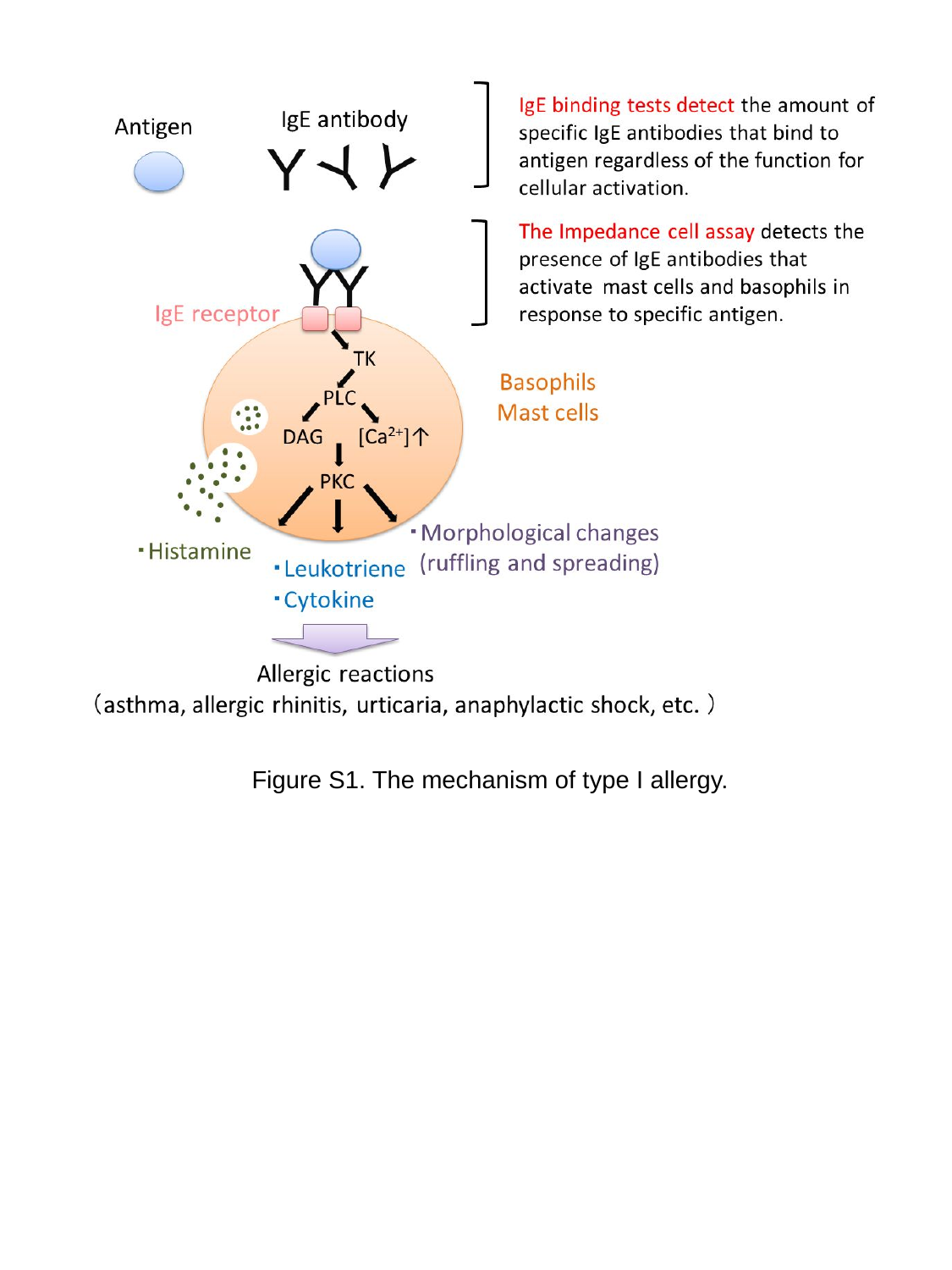

Figure S1. The mechanism of type I allergy.

## Slide 2
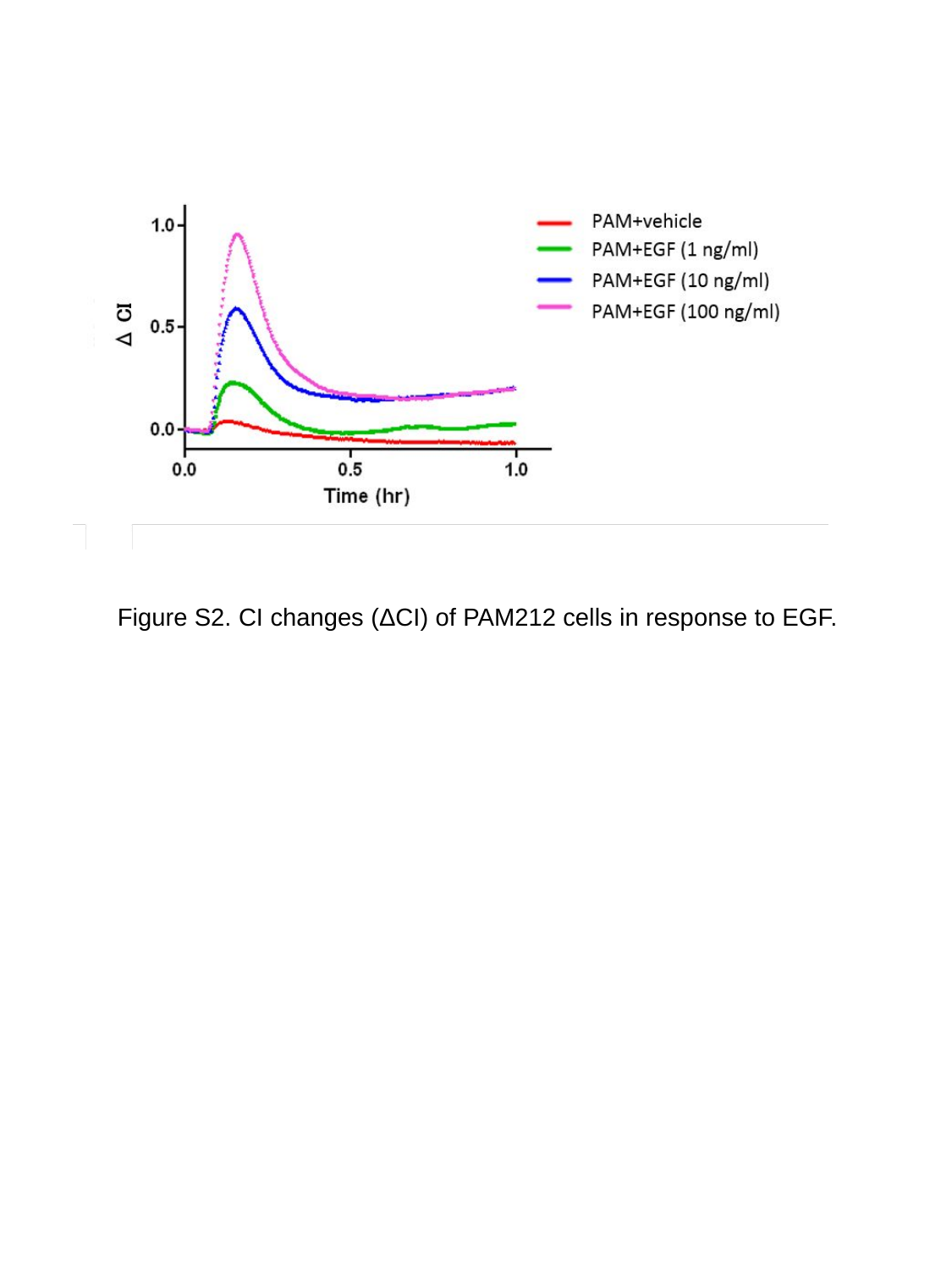

Figure S2. CI changes (ΔCI) of PAM212 cells in response to EGF.

## Slide 3
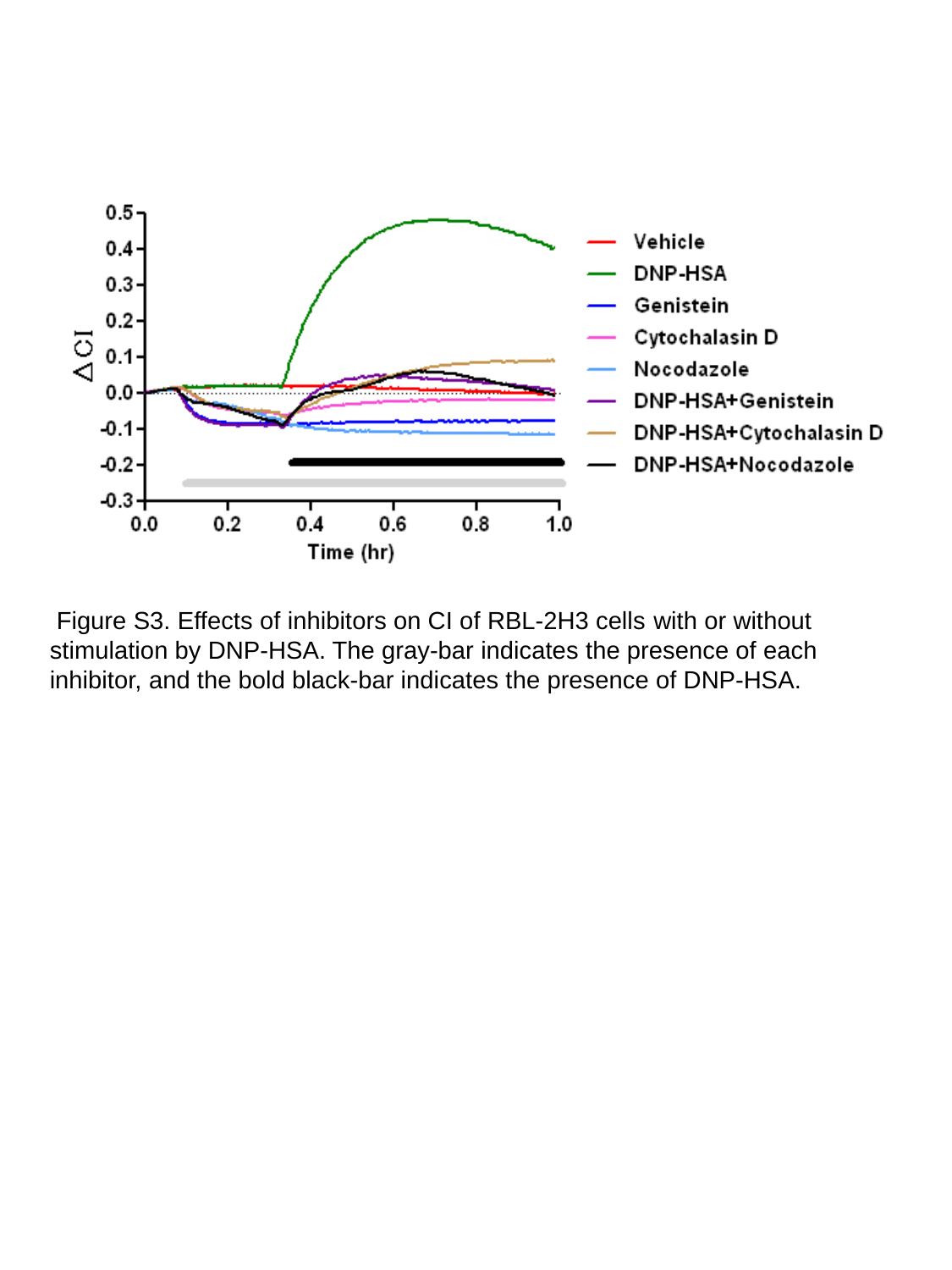

Figure S3. Effects of inhibitors on CI of RBL-2H3 cells with or without stimulation by DNP-HSA. The gray-bar indicates the presence of each inhibitor, and the bold black-bar indicates the presence of DNP-HSA.

## Slide 4
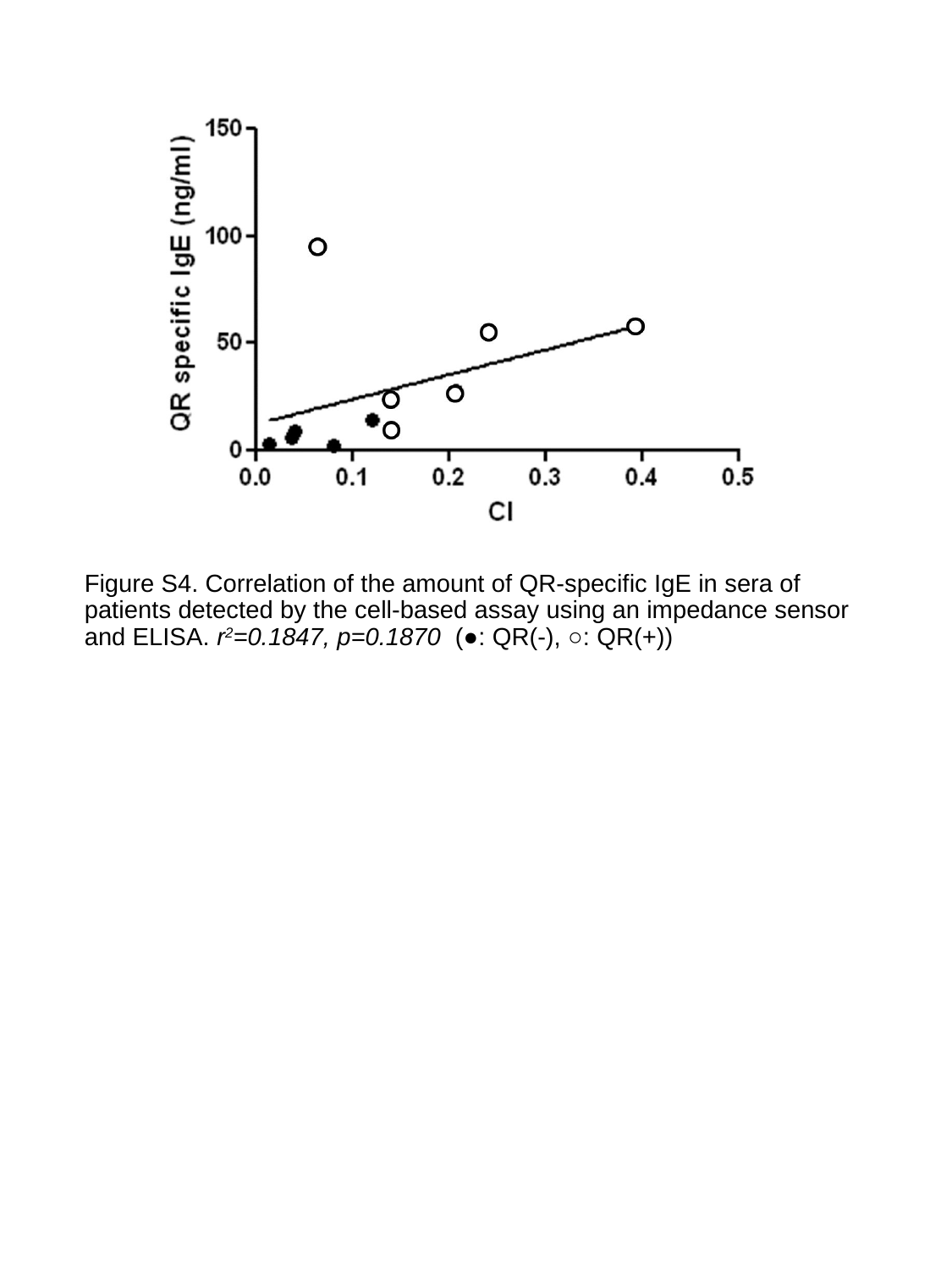

Figure S4. Correlation of the amount of QR-specific IgE in sera of patients detected by the cell-based assay using an impedance sensor and ELISA. r2=0.1847, p=0.1870 (●: QR(-), ○: QR(+))

## Slide 5
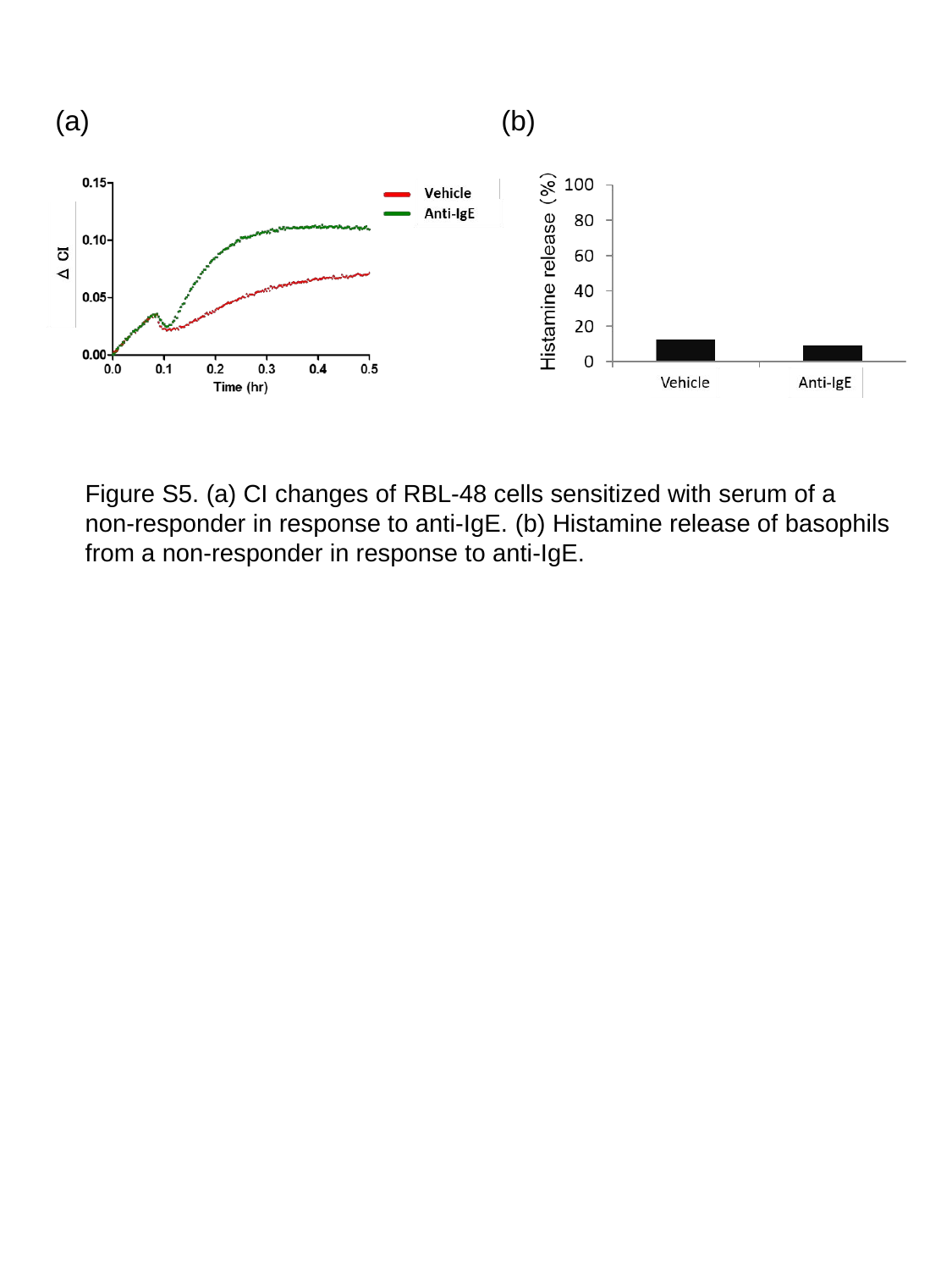

(a)
(b)
Figure S5. (a) CI changes of RBL-48 cells sensitized with serum of a non-responder in response to anti-IgE. (b) Histamine release of basophils from a non-responder in response to anti-IgE.
